# Fifty psychological and psychiatric terms to avoid: a list of inaccurate, misleading, misused, ambiguous, and logically confused words and phrases

**DOI:** 10.3389/fpsyg.2015.01100

**Published:** 2015-08-03

**Authors:** Scott O. Lilienfeld, Katheryn C. Sauvigné, Steven Jay Lynn, Robin L. Cautin, Robert D. Latzman, Irwin D. Waldman

**Affiliations:** ^1^Department of Psychology, Emory University, Atlanta, GAUSA; ^2^Department of Psychology, Georgia State University, Atlanta, GAUSA; ^3^Binghamton University – State University of New York, Binghamton, NYUSA; ^4^Department of Psychology, Sacred Heart College, Fairfield, CTUSA

**Keywords:** scientific thinking, misconceptions, misunderstandings, terminology, jingle and jangle fallacies

## Abstract

The goal of this article is to promote clear thinking and clear writing among students and teachers of psychological science by curbing terminological misinformation and confusion. To this end, we present a provisional list of 50 commonly used terms in psychology, psychiatry, and allied fields that should be avoided, or at most used sparingly and with explicit caveats. We provide corrective information for students, instructors, and researchers regarding these terms, which we organize for expository purposes into five categories: inaccurate or misleading terms, frequently misused terms, ambiguous terms, oxymorons, and pleonasms. For each term, we (a) explain why it is problematic, (b) delineate one or more examples of its misuse, and (c) when pertinent, offer recommendations for preferable terms. By being more judicious in their use of terminology, psychologists and psychiatrists can foster clearer thinking in their students and the field at large regarding mental phenomena.

“If names be not correct, language is not in accordance with the truth of things.”

(Confucius, *The Analects*)

Scientific thinking necessitates clarity, including clarity in writing (Pinker, 2014). In turn, clarity hinges on accuracy in the use of specialized terminology. Clarity is especially critical in such disciplines as psychology and psychiatry, where most phenomena, such as emotions, personality traits, and mental disorders, are “open concepts.” Open concepts are characterized by fuzzy boundaries, an indefinitely extendable indicator list, and an unclear inner essence (Pap, 1958; Meehl, 1986).

Many writers, including students, may take the inherent murkiness of many psychological and psychiatric constructs as an implicit license for looseness in language. After all, if the core concepts within a field are themselves ambiguous, the reasoning goes, precision in language may not be essential. In fact, the opposite is true; the inherent openness of many psychological concepts renders it all the more imperative that we insist on rigor in our writing and thinking to avoid misunderstandings (Guze, 1970). Researchers, teachers, and students in psychology and allied fields should therefore be as explicit as possible about what are they are saying and are *not* saying, as terms in these disciplines readily lend themselves to confusion and misinterpretation.

For at least two reasons, issues of terminology bear crucial implications for the education of forthcoming generations of students in psychology, psychiatry, and related domains. First, many instructors may inadvertently disseminate misinformation or foster unclear thinking by using specialized terms in inaccurate, vague, or idiosyncratic ways. Six decades ago, two prominent psychiatrists bemoaned the tendency of writers to use “jargon to blur implausible concepts and to convey the impression that something real is being disclosed” (Cleckley and Thigpen, 1955, p. 335). We hope that our article offers a friendly, albeit greatly belated, corrective in this regard. Second, if students are allowed, or worse, encouraged, to be imprecise in their language concerning psychological concepts, their thinking about these concepts is likely to follow suit. An insistence on clarity in language forces students to think more deeply and carefully about psychological phenomena, and serves as a potent antidote against intellectual laziness, which can substitute for the meticulous analysis of concepts. The accurate use of terminology is therefore a prerequisite to clear thinking within psychology and related disciplines.

Psychology has long struggled with problems of terminology (Stanovich, 2012). For example, numerous scholars have warned of the *jingle* and *jangle* fallacies, the former being the error of referring to different constructs by the same name and the latter the error of referring to the same construct by different names (Kelley, 1927; Block, 1995; Markon, 2009). As an example of the jingle fallacy, many authors use the term “anxiety” to refer interchangeably to trait anxiety and trait fear. Nevertheless, research consistently shows that fear and anxiety are etiologically separable dispositions and that measures of these constructs are only modestly correlated (Sylvers et al., 2011). As an example of the jangle fallacy, dozens of studies in the 1960s focused on the correlates of the ostensibly distinct personality dimension of repression-sensitization (e.g., Byrne, 1964). Nevertheless, research eventually demonstrated that this dimension was essentially identical to trait anxiety (Watson and Clark, 1984). In the field of social psychology, Hagger (2014) similarly referred to the “deja variable” problem, the ahistorical tendency of researchers to concoct new labels for phenomena that have long been described using other terminology (e.g., the use of 15 different terms to describe the false consensus effect; see Miller and Pedersen, 1999).

In this article, we present a provisional list of 50 commonly used terms in psychology, psychiatry, and allied fields that should be avoided, or at most used sparingly and with explicit caveats. For each term, we (a) explain why it is problematic, (b) delineate one or more examples of its misuse, and (c) when pertinent, offer recommendations for preferable terms. These terms span numerous topical areas within psychology and psychiatry, including neuroscience, genetics, statistics, and clinical, social, cognitive, and forensic psychology. Still, in proposing these 50 terms, we make no pretense at comprehensiveness. We are certain that many readers will have candidates for their own “least favorite” psychological and psychiatric terms, and we encourage them to contact us with their nominees. In addition, we do not include commonly confused terms (e.g., “asocial” with “antisocial,” “external validity” with “ecological validity,” “negative reinforcement” with “punishment,” “mass murderer” with ‘serial killer’), as we intend to present a list of these term pairs in a forthcoming publication. We also do not address problematic terms that are restricted primarily to popular (“pop”) psychology, such as “codependency,” “dysfunctional,” “toxic,” “inner child,” and “boundaries,” as our principal focus is on questionable terminology in the academic literature. Nevertheless, we touch on a handful of pop psychology terms (e.g., closure, splitting) that have migrated into at least some academic domains.

Our “eyeball cluster analysis” of these 50 terms has led us to group them into five overarching and partly overlapping categories for expository purposes: inaccurate or misleading terms, frequently misused terms, ambiguous terms, oxymorons, and pleonasms. Terms in all five categories, we contend, have frequently sown the seeds of confusion in psychology, psychiatry, and related fields, and in so doing have potentially impeded (a) their scientific progress and (b) clear thinking among students.

First, some psychological terms are *inaccurate* or *misleading*. For example, the term “hard-wired” as applied to human traits implies that genes rigidly prescribe complex psychological behaviors (e.g., physical aggression) and traits (e.g., extraversion), which is almost never the case. Second, some psychological terms are not incorrect *per se*, but are *frequently misused*. For example, although “splitting” carries a specific meaning as a defensive reaction in psychodynamic theory, it is commonly misused to refer to the propensity of people with borderline personality disorder (BPD) and related conditions to pit staff members against each other. Third, some psychological terms are *ambiguous*, because they can mean several things. For example, the term “medical model” can refer to any one (or more) of at least seven conceptual models of mental illness and its treatment. Fourth, some psychological terms are *oxymorons*. An oxymoron is a term, such as open secret, precise estimate, or final draft, which consists of two conjoined terms that are contradictory. For example, the term “stepwise hierarchical regression” is an oxymoron because stepwise and hierarchical multiple regression are incompatible statistical procedures. Fifth, some psychological terms are *pleonasms*. A pleonasm is a term, such as PIN number, Xerox copy, or advance warning, which consists of two or more conjoined terms that are redundant. For example, the term “latent construct” is a pleonasm because all psychological constructs are hypothetical and therefore unobservable.

Our list of 50 terms, grouped into the five aforementioned categories and presented in alphabetical order within each category, follows.

## Inaccurate or Misleading Terms

**(1) A gene for**. The news media is awash in reports of identifying “genes for” a myriad of phenotypes, including personality traits, mental illnesses, homosexuality, and political attitudes (Sapolsky, 1997). For example, in 2010, *The Telegraph (2010)* trumpeted the headline, “‘Liberal gene’ discovered by scientists.” Nevertheless, because genes code for proteins, there are no “genes for” phenotypes *per se*, including behavioral phenotypes (Falk, 2014). Moreover, genome-wide association studies of major psychiatric disorders, such as schizophrenia and bipolar disorder, suggest that there are probably few or no genes of major effect (Kendler, 2005). In this respect, these disorders are unlike single-gene medical disorders, such as Huntington’s disease or cystic fibrosis. The same conclusion probably holds for all personality traits (De Moor et al., 2012).

Not surprisingly, early claims that the monoamine oxidase-A (MAO-A) gene is a “warrior gene” (McDermott et al., 2009) have not withstood scrutiny. This polymorphism appears to be only modestly associated with risk for aggression, and it has been reported to be associated with conditions that are not tied to a markedly heightened risk of aggression, such as major depression, panic disorder, and autism spectrum disorder (Buckholtz and Meyer-Lindenberg, 2013; Ficks and Waldman, 2014). The evidence for a “God gene,” which supposedly predisposes people to mystical or spiritual experiences, is arguably even less impressive (Shermer, 2015) and no more compelling than that for a “God spot” in the brain (see “God spot”). Incidentally, the term “gene” should not be confused with the term “allele”; genes are stretches of DNA that code for a given morphological or behavioral characteristic, whereas alleles are differing versions of a specific polymorphism in a gene (Pashley, 1994).

**(2) Antidepressant medication**. Medications such as tricyclics, selective serotonin reuptake inhibitors, and selective serotonin and norepinephrine reuptake inhibitors, are routinely called “antidepressants.” Yet there is little evidence that these medications are more efficacious for treating (or preventing relapse for) mood disorders than for several other conditions, such as anxiety-related disorders (e.g., panic disorder, obsessive-compulsive disorder; Donovan et al., 2010) or bulimia nervosa (Tortorella et al., 2014). Hence, their specificity to depression is doubtful, and their name derives more from historical precedence—the initial evidence for their efficacy stemmed from research on depression (France et al., 2007)—than from scientific evidence. Moreover, some authors argue that these medications are considerably less efficacious than commonly claimed, and are beneficial for only severe, but not mild or moderate, depression, rendering the label of “antidepressant” potentially misleading (Antonuccio and Healy, 2012; but see Kramer, 2011, for an alternative view).

**(3) Autism epidemic**. Enormous effort has been expended to uncover the sources of the “autism epidemic” (e.g., King, 2011), the supposed massive increase in the incidence and prevalence of autism, now termed autism spectrum disorder, over the past 25 years. The causal factors posited to be implicated in this “epidemic” have included vaccines, television viewing, dietary allergies, antibiotics, and viruses.

Nevertheless, there is meager evidence that this purported epidemic reflects a genuine increase in the rates of autism *per se* as opposed to an increase in autism *diagnoses* stemming from several biases and artifacts, including heightened societal awareness of the features of autism (“detection bias”), growing incentives for school districts to report autism diagnoses, and a lowering of the diagnostic thresholds for autism across successive editions of the Diagnostic and Statistical Manual of Mental Disorders (Gernsbacher et al., 2005; Lilienfeld and Arkowitz, 2007). Indeed, data indicate when the diagnostic criteria for autism were held constant, the rates of this disorder remained essentially constant between 1990 and 2010 (Baxter et al., 2015). If the rates of autism are increasing, the increase would appear to be slight at best, hardly justifying the widespread claim of an “epidemic.”

**(4) Brain region X lights up**. Many authors in the popular and academic literatures use such phrases as “brain area X lit up following manipulation Y” (e.g., Morin, 2011). This phrase is unfortunate for several reasons. First, the bright red and orange colors seen on functional brain imaging scans are superimposed by researchers to reflect regions of higher brain activation. Nevertheless, they may engender a perception of “illumination” in viewers. Second, the activations represented by these colors do not reflect neural activity *per se*; they reflect oxygen uptake by neurons and are at best indirect proxies of brain activity. Even then, this linkage may sometimes be unclear or perhaps absent (Ekstrom, 2010). Third, in almost all cases, the activations observed on brain scans are the products of *subtraction* of one experimental condition from another. Hence, they typically do not reflect the raw levels of neural activation in response to an experimental manipulation. For this reason, referring to a brain region that displays little or no activation in response to an experimental manipulation as a “dead zone” (e.g., Lamont, 2008) is similarly misleading. Fourth, depending on the neurotransmitters released and the brain areas in which they are released, the regions that are “activated” in a brain scan may actually be being inhibited rather than excited (Satel and Lilienfeld, 2013). Hence, from a functional perspective, these areas may be being “lit down” rather than “lit up.”

**(5) Brainwashing**. This term, which originated during the Korean War (Hunter, 1951) but which is still invoked uncritically from time to time in the academic literature (e.g., Ventegodt et al., 2009; Kluft, 2011), implies that powerful individuals wishing to persuade others can capitalize on a unique armamentarium of coercive procedures to change their long-term attitudes. Nevertheless, the attitude-change techniques used by so-called “brainwashers” are no different than standard persuasive methods identified by social psychologists, such as encouraging commitment to goals, manufacturing source credibility, forging an illusion of group consensus, and vivid testimonials (Zimbardo, 1997). Furthermore, there are ample reasons to doubt whether “brainwashing” permanently alters beliefs (Melton, 1999). For example, during the Korean War, only a small minority of the 3500 American political prisoners subjected to intense indoctrination techniques by Chinese captors generated false confessions. Moreover, an even smaller number (probably under 1%) displayed any signs of adherence to Communist ideologies following their return to the US, and even these were individuals who returned to Communist subcultures (Spanos, 1996).

**(6) Bystander apathy**. The classic work of (e.g., Darley and Latane, 1968; Latane and Rodin, 1969) underscored the counterintuitive point that when it comes to emergencies, there is rarely “safety in numbers.” As this and subsequent research demonstrated, the more people present at an emergency, the lower the likelihood of receiving help. In early research, this phenomenon was called “bystander apathy” (Latane and Darley, 1969) a term that endures in many academic articles (e.g., Abbate et al., 2013). Nevertheless, research demonstrates that most bystanders are far from apathetic in emergencies (Glassman and Hadad, 2008). To the contrary, they are typically quite concerned about the victim, but are psychologically “frozen” by well-established psychological processes, such as pluralistic ignorance, diffusion of responsibility, and sheer fears of appearing foolish.

**(7) Chemical imbalance**. Thanks in part to the success of direct-to-consumer marketing campaigns by drug companies, the notion that major depression and allied disorders are caused by a “chemical imbalance” of neurotransmitters, such as serotonin and norepinephrine, has become a virtual truism in the eyes of the public (France et al., 2007; Deacon and Baird, 2009). This phrase even crops up in some academic sources; for example, one author wrote that one overarching framework for conceptualizing mental illness is a “biophysical model that posits a chemical imbalance” (Wheeler, 2011, p. 151). Nevertheless, the evidence for the chemical imbalance model is at best slim (Lacasse and Leo, 2005; Leo and Lacasse, 2008). One prominent psychiatrist even dubbed it an urban legend (Pies, 2011). There is no known “optimal” level of neurotransmitters in the brain, so it is unclear what would constitute an “imbalance.” Nor is there evidence for an optimal ratio among different neurotransmitter levels. Moreover, although serotonin reuptake inhibitors, such as fluoxetine (Prozac) and sertraline (Zoloft), appear to alleviate the symptoms of severe depression, there is evidence that at least one serotonin reuptake *enhancer*, namely tianepine (Stablon), is also efficacious for depression (Akiki, 2014). The fact that two efficacious classes of medications exert opposing effects on serotonin levels raises questions concerning a simplistic chemical imbalance model.

**(8) Family genetic studies**. The phrase “family genetic studies” is commonly used in psychiatry to refer to designs in which investigators examine the familial aggregation of one or more disorders, such as panic disorder or major depression, within intact (i.e., non-adoptive) families (e.g., Weissman, 1993). Given that the familial aggregation of one or more disorders within intact families could be due to shared environment rather than—or in addition to—shared genes (Smoller and Finn, 2003), the phrase “family genetic study” is misleading. This term implies erroneously that familial clustering of a disorder is necessarily more likely to be genetic than environmental. It may also imply incorrectly (Kendler and Neale, 2009) that studies of intact families permit investigators to disentangle the effects of shared genes from shared environment. Twin or adoption studies are necessary to accomplish this goal.

**(9) Genetically determined**. Few if any psychological capacities are genetically “determined”; at most, they are genetically influenced. Even schizophrenia, which is among the most heritable of all mental disorders, appears to have a heritability of between 70 and 90% as estimated by twin designs (Mulle, 2012), leaving room for still undetermined environmental influences. Moreover, data strongly suggest that schizophrenia and most other major mental disorders are highly polygenic. In addition, the heritability of most adult personality traits, such as neuroticism and extraversion, appears to be between 30 and 60% (Kandler, 2012). This finding again points to a potent role for environmental influences.

**(10) God spot**. Seizing on functional imaging findings that religious ideation is associated with activations in specific brain regions, such as circumscribed areas of the temporal lobe, some media and academic sources have referred to the discovery of a “God spot” in the human brain (Connor, 1997). Such language is scientifically dubious given that complex psychological capacities, including religious experiences, are almost surely distributed across several sprawling networks that themselves encompass multiple brain regions. Not surprisingly, studies of people undergoing mystical experiences have reported activation in many brain areas, including the temporal lobe, caudate, inferior parietal lobe, and insula (Beauregard and Paquette, 2006; Jarrett, 2014). As one researcher (Mario Beauregard) observed, “There is no single God spot localized uniquely in the temporal lobe of the human brain” (Biello, 2007, p. 43). The same absence of localizational specificity holds for claims regarding the identification of other purported brain regions, such as an “irony spot” or “humor spot” (Jarrett, 2014).

**(11) Gold standard**. In the domains of psychological and psychiatric assessment, there are precious few, if any, genuine “gold standards.” Essentially all measures, even those with high levels of validity for their intended purposes, are necessarily fallible indicators of their respective constructs (Cronbach and Meehl, 1955; Faraone and Tsuang, 1994). As a consequence, the widespread practice referring to even well-validated measures of personality or psychopathology, such as Hare’s (1991/2003) Psychopathy Checklist-Revised, as “gold standards” for their respective constructs (Ermer et al., 2012) is misleading (see Skeem and Cooke, 2010). If authors intend to refer to measures as “extensively validated,” they should simply do so.

**(12) Hard-wired**. The term “hard-wired” has become enormously popular in press accounts and academic writings in reference to human psychological capacities that are presumed by some scholars to be partially innate, such as religion, cognitive biases, prejudice, or aggression. For example, one author team reported that males are more sensitive than females to negative news stories and conjectured that males may be “hard wired for negative news” (Grabe and Kamhawi, 2006, p. 346). Nevertheless, growing data on neural plasticity suggest that, with the possible exception of inborn reflexes, remarkably few psychological capacities in humans are genuinely hard-wired, that is, inflexible in their behavioral expression (Huttenlocher, 2009; Shermer, 2015). Moreover, virtually all psychological capacities, including emotions and language, are modifiable by environmental experiences (Merzenich, 2013).

**(13) Hypnotic trance**. The notion that hypnosis is characterized by a distinct “trance state” remains one of the enduring myths of popular psychology (Lilienfeld et al., 2009). In a sample of 276 undergraduates, Green (2003; see also Green et al., 2006) found that participants gave high ratings (between 5 and 5.5 on 1–7 scale in two experimental conditions) to the item, “Hypnosis is an altered state of consciousness, quite different from normal waking consciousness” (p. 373). Perhaps not surprisingly, the phrase “hypnotic trance” continues to appear in numerous articles written for the general public (Brody, 2008) as well as in academic sources (Raz, 2011). Nevertheless, the evidence that hypnosis is a distinct “trance” state that differs qualitatively from waking consciousness is scant. There is no consistent evidence for distinctive physiological (e.g., functional brain imaging) markers of hypnosis (Lynn et al., 2007). Nor is there persuasive, or even especially suggestive, evidence that hypnosis is associated with unique behavioral features. For example, suggested responses, including hallucinations, amnesia, and pain reduction, can be achieved in the absence of a “hypnotic induction” and even when participants report being awake and alert (Lynn et al., 2015).

**(14) Influence of gender (or social class, education, ethnicity, depression, extraversion, intelligence, etc.) on X.** “Influence” and cognate terms, such as effect, are inherently causal in nature. Hence, they should be used extremely judiciously in reference to individual differences, such as personality traits (e.g., extraversion), or group differences (e.g., gender), which cannot be experimentally manipulated. This is not to say that individual or group differences cannot exert a causal influence on behavior (Funder, 1991), only that research designs that examine these differences are virtually always (with the rare exception of “experiments of nature,” in which individual differences are altered by unusual events) correlation or quasi-experimental. Hence, researchers should be explicit that when using such phrases as “the influence of gender,” they are almost always proposing a hypothesis from the data, not drawing a logically justified conclusion from them. This inferential limitation notwithstanding, the phrase “the influence of gender” alone appears in over 45,000 manuscripts in the *Google Scholar* database (e.g., Bertakis et al., 1995).

**(15) Lie detector test**. Surely one of the most pernicious misnomers in psychology, the term “lie detector test” is often used synonymously with the storied polygraph test. This test is misnamed: it is an arousal detector, not a lie detector (Saxe et al., 1985). Because it measures non-specific psychophysiological arousal rather than the fear of detection *per se*, it is associated with high false-positive rates, meaning that it frequently misidentifies honest individuals as dishonest (Lykken, 1998). In addition, the polygraph test is susceptible to false-negatives stemming from the use of physical (e.g., biting the tongue) and mental (e.g., performing complex mental arithmetic) countermeasures (Honts et al., 1994). This evidence notwithstanding, the mythical allure of the polygraph test persists. In one survey, 45% of undergraduates agreed that this test is an accurate detector of falsehoods (Taylor and Kowalski, 2010).

**(16) Love molecule**. Over 6000 websites have dubbed the hormone oxytocin the “love molecule” (e.g., Morse, 2011). Others have named it the “trust molecule” (Dvorsky, 2012), “cuddle hormone” (Griffiths, 2014), or “moral molecule” (Zak, 2013). Nevertheless, data derived from controlled studies imply that all of these appellations are woefully simplistic (Wong, 2012; Jarrett, 2015; Shen, 2015). Most evidence suggests that oxytocin renders individuals more sensitive to social information (Stix, 2014), both positive and negative. For example, although intranasal oxytocin seems to increase within-group trust, it may also increase out-group mistrust (Bethlehem et al., 2014). In addition, among individuals with high levels of trait aggressiveness, oxytocin boosts propensities toward intimate partner violence following provocation (DeWall et al., 2014). Comparable phrases applied to other neural messengers, such as the term “pleasure molecule” as a moniker for dopamine, are equally misleading (see Landau et al., 2008; Kringelbach and Berridge, 2010, for discussions).

**(17) Multiple personality disorder**. Although the term “multiple personality disorder” was expunged from the American Psychiatric Association’s (1994) diagnostic manual over two decades ago and has since been replaced by “dissociative identity disorder” (DID), it persists in many academic sources (e.g., Hayes, 2014). Nevertheless, even ardent proponents of the view that DID is a naturally occurring condition that stems largely from childhood trauma (e.g., Ross, 1994) acknowledge that “multiple personality disorder” is a misnomer (Lilienfeld and Lynn, 2015), because individuals with DID do not genuinely harbor two or more fully developed personalities. Moreover, laboratory studies of the memories of individuals with DID demonstrate that the “alter” personalities or personality states of individuals with DID are not insulated by impenetrable amnestic barriers (Merckelbach et al., 2002).

**(18) Neural signature**. One group of authors, after observing that compliance with social norms was associated with activations in certain brain regions (lateral orbitofrontal cortex and right dorsolateral cortex), referred to the “neural signature” of social norm compliance (Spitzer et al., 2007, p. 185). Others have referred to neural signatures or “brain signatures” of psychiatric disorders, such as anorexia nervosa (Fladung et al., 2009) and autism spectrum disorder (Pelphrey and McPartland, 2012). Nevertheless, identifying a genuine neural signature would necessitate the discovery of a specific pattern of brain responses that possesses nearly perfect sensitivity and specificity for a given condition or other phenotype. At the present time, neuroscientists are not remotely close to pinpointing such a signature for any psychological disorder or trait (Gillihan and Parens, 2011).

**(19) No difference between groups**. Many researchers, after reporting a group difference that does not attain conventional levels of statistical significance, will go on to state that “there was no difference between groups.” Similarly, many authors will report that a non-significant correlation between two variables means that “there was no association between the variables.” But a failure to reject the null hypothesis does not mean that the null hypothesis, strictly speaking, has been confirmed. Indeed, if an investigator finds a correlation of *r* = 0.11 in a sample of 20 participants (which is not statistically significant), the best estimate for the true value of the correlation in the population, presuming that the sample has been randomly ascertained, is 0.11, not 0. Authors are instead advised to write “no significant difference between groups” or “no significant correlation between variables.”

**(20) Objective personality test.** Many authors refer to paper-and-pencil personality instruments that employ a standard (e.g., True–False) item response format, such as the Minnesota Multiphasic Personality Inventory-2 (MMPI-2), as “objective tests” (Proyer and Häusler, 2007), ostensibly to contrast them with more “subjective” measures, such as unstructured interviews or projective techniques (e.g., the Rorschach Inkblot Test). Nevertheless, although the former measures can be scored objectively, that is, with little or no error (but see Allard and Faust, 2000, for evidence of non-trivial error rates in the hand-scoring of the MMPI and other purported “objective” personality tests), they often require considerable subjective judgment on the part of respondents. For example, an item such as “I have many headaches” can be interpreted in numerous ways arising from ambiguity in the meanings of “many” and “headache’ (Meehl, 1945). So-called “objective” personality tests are also often subjective with respect to interpretation (Rogers, 2003). For example, even different computerized MMPI-2 interpretive programs display only moderate levels of inter-rater agreement regarding proposed diagnoses (Pant et al., 2014). Not surprisingly, clinicians routinely disagree in their interpretations of profiles on the MMPI-2 and other “objective” tests (Garb, 1998). We therefore recommend that these measures be called “structured” tests (Kaplan and Saccuzzo, 2012), a term that refers only to their response format and that carries no implication that they are interpreted objectively by either examinee or examiner.

**(21) Operational definition**. The credo that all psychological investigators must develop “operational definitions” of constructs before conducting studies has become something of a truism in many psychology methods textbooks and other research sources (e.g., Burnette, 2007). Operational definitions are strict definitions of concepts in terms of their measurement operations. As a consequence, they are presumed to be exact and exhaustive definitions of these concepts. Perhaps the best known example in psychology is Boring’s (1923) definition of intelligence as whatever intelligence tests measure.

Many psychologists appear unaware that the notion of operational definitions was roundly rejected by philosophers of science decades ago (Leahey, 1980; Green, 1992; Gravetter and Forzano, 2012). Operational definitions are unrealistic in virtually all domains of psychology, because constructs are not equivalent to their measurement operations (Meehl, 1986). For example, an “operational definition” of aggression as the amount of hot sauce a participant places in an experimental confederate’s drink is not an operational definition at all, because no researcher seriously believes that the amount of hot sauce placed in a drink is a perfect or precise definition of aggression that exhausts all of its potential manifestations. Operational definitions also fell out of favor because they led to logically absurd conclusions. For example, an operational definition of length would imply that length as measured by a wooden ruler cannot be compared with length as measured by a metal ruler, because these rulers are associated with different measurement operations. Hence, the fact that both rulers yield a length for a table of say, 27 inches, could not be taken as converging evidence that the table is in fact 27 inches long (Green, 1992).

Psychological researchers and teachers should therefore almost always steer clear of the term “operational definition.” The term “operationalization” is superior, as it avoids the implication of an ironclad definition and is largely free of the problematic logical baggage associated with its sister term.

**(22) *p* = 0.000**. Even though this statistical expression, used in over 97,000 manuscripts according to *Google Scholar*, makes regular cameo appearances in our computer printouts, we should assiduously avoid inserting it in our *Results* sections. This expression implies erroneously that there is a *zero* probability that the investigators have committed a Type I error, that is, a false rejection of a true null hypothesis (Streiner, 2007). That conclusion is logically absurd, because unless one has examined essentially the entire population, there is always some chance of a Type I error, no matter how meager. Needless to say, the expression “*p* < 0.000” is even worse, as the probability of committing a Type I error cannot be less than zero. Authors whose computer printouts yield significance levels of *p* = 0.000 should instead express these levels out to a large number of decimal places, or at least indicate that the probability level is below a given value, such as *p* < 0.01 or *p* < 0.001.

**(23) Psychiatric control group**. This phrase and similar phrases (e.g., “normal control group,” “psychopathological control group”) connote erroneously that (a) groups of ostensibly normal individuals or mixed psychiatric patients who are being compared with (b) groups of individuals with a disorder of interest (e.g., schizophrenia, major depression) are true “control” groups. They are not. They are “comparison groups” and should be referred to accordingly. The phrase “control group” in this context may leave readers with the unwarranted impression that the design of the study is experimental when it is actually quasi-experimental. Just as important, this term may imply that the only difference between the two groups (e.g., a group of patients with anxiety disorder and a group of ostensibly normal individuals) is the presence or absence of the disorder of interest. In fact, these two groups almost surely differ on any number of “nuisance” variables, such as personality traits, co-occurring disorders, and family background, rendering the interpretation of most group differences open to multiple interpretations (Meehl, 1969).

**(24) Reliable and valid**. If one earned a dollar for every time an author used the sentence “This test is reliable and valid” in a *Method* section, one would be a rich person indeed, as the phrase “reliable and valid” appears in more than 190,000 manuscripts in *Google Scholar*. There are at least three problems with this ubiquitous phrase. First, it implies that a psychological test is either valid or not valid. Much like the testing of scientific theories, the construct validation process is never complete, in essence reflecting a “work in progress.” As a consequence, a test cannot be said to be have been conclusively validated or invalidated (Cronbach and Meehl, 1955; Loevinger, 1957; Peter, 1981). Hence, authors should similarly refrain from using the term “validated’ with respect to psychological measures. At best, these measures are “empirically supported” or have “accrued substantial evidence for construct validity.” The same caveat applies to psychological treatments. When Division 12 (Society of Clinical Psychology) of the American Psychological Association put forth its criteria for, and lists of, psychotherapies found to work in controlled trials for specific mental disorders, it initially termed them “empirically validated therapies” (Chambless et al., 1998). Nevertheless, in recognition of the fact that “validation” implies certainty or finality (Garfield, 1996; Chambless and Hollon, 1998), the committee wisely changed the name to “empirically supported therapies,” which is now the term presently in use (Lilienfeld et al., 2013).

Second, the phrase “reliable and valid” implies that reliability and validity are unitary concepts. They are not. There are three major forms of reliability: test–retest, internal consistency, and inter-rater. Contrary to common belief, these forms of reliability often diverge, sometimes markedly (Schmidt and Hunter, 1996). For example, scores derived from the Thematic Apperception Test, a widely used projective technique, frequently display high levels of test–retest reliability but low levels of internal consistency (Entwistle, 1972). There are also multiple forms of validity (e.g., content, criterion-related, incremental), which similarly do not necessarily coincide. For example, a measure may possess high levels of criterion-related validity in multiple samples but little or no incremental validity above and beyond extant information (Garb, 2003).

Third, reliability and validity are conditional on the specific samples examined, and should not be considered inherent properties of a test. Hence, the notion that a test is “reliable and valid” independent of the nature of the sample runs counter to contemporary thinking in psychometrics (American Psychological Association and American Educational Research Association, 2014).

**(25) Statistically reliable**. This phrase appears in over 62,000 manuscripts according to *Google Scholar*. It is typically invoked when referring to statistical significance, e.g., “Although small in absolute terms, this difference was statistically reliable, *t*(157) = 2.86, *p* = 0.005” (Zurbriggen et al., 2011, p. 453). Nevertheless, despite what many psychologists believe (Tversky and Kahneman, 1971; Krueger, 2001), statistical significance bears at best a modest conceptual and empirical association with a result’s “reliability,” that is, its replicability or consistency over time (Carver, 1978). Indeed, given the low statistical power of most studies in psychology, a reasonable argument could be advanced that most statistically significant results are unlikely to be reliable. The statistical significance of a result should therefore not be confused with its likelihood of replication (Miller, 2009).

**(26) Steep learning curve**. Scores of authors use the phrase “steep learning curve” or “sharp learning curve” in reference to a skill that is difficult to master. For example, when referring to the difficulty of learning a complex surgical procedure (endoscopic pituitary surgery), one author team contended that it “requires a steep learning curve” (Koc et al., 2006, p. 299). Nevertheless, from the standpoint of learning theory, these and other authors have it backward, because a steep learning curve, i.e., a curve with a large positive slope, is associated with a skill that is acquired easily and rapidly (Hopper et al., 2007).

**(27) The scientific method**. Many science textbooks, including those in psychology, present science as a monolithic “method.” Most often, they describe this method as a hypothetical-deductive recipe, in which scientists begin with an overarching theory, deduce hypotheses (predictions) from that theory, test these hypotheses, and examine the fit between data and theory. If the data are inconsistent with the theory, the theory is modified or abandoned. It’s a nice story, but it rarely works this way (McComas, 1996). Although science sometimes operates by straightforward deduction, serendipity and inductive observations offered in the service of the “context of discovery” also play crucial roles in science. For this reason, the eminent philosopher of science Popper (1983) quipped that, “As a rule, I begin my lectures on Scientific Method by telling my students that the scientific method does not exist…” (p. 5).

Contrary to what most scientists themselves appear to believe, science is *not* a method; it is an approach to knowledge (Stanovich, 2012). Specifically, it is an approach that strives to better approximate the state of nature by reducing errors in inferences. Alternatively, one can conceptualize science as a toolbox of finely honed tools designed to minimize mistakes, especially confirmation bias – the ubiquitous propensity to seek out and selectively interpret evidence consistent with our hypotheses and to deny, dismiss, and distort evidence that does not (Tavris and Aronson, 2007; Lilienfeld, 2010). Not surprisingly, the specific research methods used by psychologists bear scant surface resemblance to those used by chemists, astrophysicists, or molecular biologists. Nevertheless, all of these methods share an overarching commitment to reducing errors in inference and thereby arriving at a more accurate understanding of reality.

**(28) Truth serum**. “Truth serum” is a supposed substance that, when administered intravenously, leads individuals to disclose accurate information that they have withheld. Most so-called truth serums are actually barbiturates, such as sodium amytal or sodium pentothal (Keller, 2005). Even today, some prominent psychiatrists still refer to these substances as truth serums (e.g., Lieberman, 2015), and they are still frequently administered for legal purposes in certain countries, such as India (Pathak and Srivastava, 2011). Nevertheless, there is no evidence that so-called truth serums reveal veridical information regarding past events, such as childhood sexual abuse (Bimmerle, 1993). To the contrary, like other suggestive memory procedures, they are associated with a heightened risk of false memories and false confessions (Macdonald, 1955), probably because they lower the response threshold for reporting all information, accurate and inaccurate alike. Furthermore, individuals can and do readily lie under the influence of truth serum (Piper, 1993).

**(29) Underlying biological dysfunction**. In this era of the increasing biologization of psychology and psychiatry (Miller, 2010; Satel and Lilienfeld, 2013), authors may be tempted to assume that biological variables, such as parameters of brain functioning, “underlie” psychological phenomena. For example, one set of authors wrote that “cognitive impairments are central to schizophrenia and may mark underlying biological dysfunction” (Bilder et al., 2011, p. 426). Nevertheless, conceptualizing biological functioning as inherently more “fundamental” than (that is, causally prior to) psychological functioning, such as cognitive and emotional functioning, is misleading (Miller, 1996). The relation between biological variables and other variables is virtually always bidirectional. For example, although the magnitude of the P300 event-related potential tends to be diminished among individuals with antisocial personality disorder (ASPD) compared with other individuals (Costa et al., 2000), this finding does not necessarily mean that the P300 deficit precedes, let alone plays a causal role in, ASPD. It is at least equally plausible that the personality dispositions associated with ASPD, such as inattention, low motivation, and poor impulse control, contribute to smaller P300 magnitudes (Lilienfeld, 2014). The same inferential limitation applies to many similar phrases, such as “biological bases of behavior,” “brain substrates of mental disorder,” and “neural underpinnings of personality” (Miller, 1996).

## Frequently Misused Terms

**(30) Acting out**. Numerous articles use this term as a synonym for any kind of externalizing or antisocial behavior, including delinquency (e.g., Weinberger and Gomes, 1995). In fact, the term “acting out” carries a specific psychoanalytic meaning that refers to the behavioral enactment of unconscious drives that are ostensibly forbidden by the superego (Fenichel, 1945). Hence, this term should not be used interchangeably with disruptive behavior of all kinds and attributable to all causes.

**(31) Closure**. The term “closure” was introduced by Gestalt psychologists (Koffka, 1922) to refer to the tendency to perceive incomplete figures as wholes. This term has since been misappropriated by popular psychologists (Howard, 2011) and social scientists of various stripes (e.g., Skitka et al., 2004) to describe the purported experience of emotional resolution experienced by victims of trauma following an event of symbolic importance. For example, many advocates of the “closure movement” contend that the execution of a murderer assists the loved ones of victims to put an end to their grieving process. Nevertheless, this use of the term “closure” is hopelessly vague, as it is rarely if ever clear when trauma victims have achieved the desired emotional end-state (Radford, 2003; Weinstein, 2011). Nor is there research support for the proposition that many or most victims experience this end-state after events of symbolic significance, such as executions or funerals (Berns, 2011).

**(32) Denial**. Denial, a psychodynamic defense mechanism popularized by Freud (1937), is an ostensibly unconscious refusal to acknowledge obvious facts of reality, such as the death of a loved one in an automobile accident (Vaillant, 1977). Nevertheless, thanks largely to the popular psychology industry, this term has been widely misappropriated to refer to the tendency of individuals with a psychological condition, such as alcohol use disorder (formerly called alcoholism), to minimize the extent of their pathology (e.g., Wing, 1995).

**(33) Fetish**. A fetish, formally referred to as “Fetishistic Disorder” in the current version of the Diagnostic and Statistical Manual of Mental Disorders (DSM-5; American Psychiatric Association, 2013, p. 700), is a psychiatric condition marked by persistent, intense, and psychologically impairing sexual arousal derived from inanimate objects (e.g., shoes) or non-genital body parts (e.g., legs). This term, which is technically a paraphilia, should not be used to refer to generic preferences for specific objects, ideas, or people. One writer, for example, described the national fascination of the Japanese with smartphones as a “feature phone fetish” (Smith, 2015).

**(34) Splitting**. “Splitting” similarly refers to a psychodynamic defense mechanism, ostensibly ubiquitous in BPD, that forces individuals to see people as all good or all bad rather than in shades of gray, warts and all (Muller, 1992). By engaging in splitting, people with BPD and similar conditions are hypothesized to avoid the anxiety of perceiving those they love as the hopelessly flawed creatures that they are. Nevertheless, this term is consistently misused to refer to the propensity of people with BPD to “pit” staff members on a psychiatric unit (or other caregivers) against one another. This disruptive behavior, sometimes termed “staff splitting” (Linehan, 1989), should not be confused with the formal meaning of splitting.

## Ambiguous Terms

**(35) Comorbidity**. This term, which has become ubiquitous in publications on the relations between two or more mental disorders (appearing in approximately 444,000 citations in *Google Scholar*), refers to the overlap between two diagnoses, such as major depression and generalized anxiety disorder. A similar term, “dual diagnosis,” which has acquired considerable currency in the substance abuse literature in particular, refers to the simultaneous presence of a mental disorder, such as schizophrenia, and a substance abuse disorder, such as alcoholism (Dixon, 1999). Some authors have taken the comorbidity concept further, extending it to “trimorbidity” (Cornelius et al., 2001) or “quatromorbidity” (Newman et al., 1998).

Nevertheless, “comorbidity” can mean two quite different things. It can refer to either the (a) covariation (or correlation) between two diagnoses *within a sample or the population* or (b) co-occurrence between two diagnoses *within an individual* (Lilienfeld et al., 1994; Krueger and Markon, 2006). The first meaning refers to the extent to which Condition A and B are statistically associated across individuals; for example, there is substantial covariation between ASPD and BPD (Becker et al., 2014). The second meaning is a conditional probability referring to the proportion of individuals with Condition A who meet diagnostic criteria for Condition B. For example, in the case of the latter meaning, researchers might note that 45% of patients with ASPD also meet diagnostic criteria for BPD. The difference between these two meanings is hardly trivial, because they tend to be differentially influenced by base rates (prevalences). If the base rates of one or more conditions change, the covariation between them will not necessarily be affected but the level of co-occurrence almost always will be (Lilienfeld et al., 1994). Moreover, depending on the base rates of the diagnoses in a sample, two conditions may display little or no covariation but substantial co-occurrence. For example, although ASPD and major depression typically display only modest covariation (Goodwin and Hamilton, 2003), the rates of co-occurrence between ASPD and major depression in an analysis conditioned on major depression (that is, the rates of ASPD among people with major depression) would be extremely high in a prison sample, because most prison inmates meet criteria for ASPD (Flint-Stevens, 1993). Hence, the levels of comorbidity would probably be negligible in the first case but high in the second. If authors elect to use the term “comorbidity,” they should therefore be explicit about which meaning (covariation or co-occurrence) they intend.

Some authors (Lilienfeld et al., 1994) have further questioned the routine use of the term comorbidity in psychopathology research given that this term, much like “dual diagnosis,” presupposes that the conditions in question are etiologically and pathologically separable entities (but see Rutter, 1994; Spitzer, 1994, for demurrals). For example, although the high level of “comorbidity” between ASPD and BPD may reflect covariation or co-occurrence between two distinct conditions, it may instead reflect the fact that the current diagnostic system is attaching different names to slightly different manifestations of a shared diathesis, thereby falling prey to a jangle fallacy. To take an admittedly extreme example, how likely is it that a participant in a published study who simultaneously met diagnostic criteria for all 10 DSM personality disorders (see Lilienfeld et al., 2013) genuinely possessed 10 distinct disorders at the same time? Critics of the expansive application of the term comorbidity to descriptive psychopathology contend that these diagnostic conundrums are best explained by a flawed diagnostic system that is attaching different names to highly overlapping constructs.

**(36) Interaction**. As Olweus (1977) observed in the context of the person-situation debate, the term “interaction” has multiple meanings, some of them logically incompatible. For example, the familiar phrase “genes and environment interact for Disorder X” can mean any one of four things: (a) genes and environment are both involved in the causes of Disorder X; (b) the relation between genes and environments are bidirectional, because genes influence the environments to which people are exposed (by means of gene-environment correlations), and environments influence which genes are activated or inactivated (by means of epigenetic processes); (c) the influences of genes and environment are inseparable because of continuous transaction within individuals; or (d) the statistical effects of genes depend on people’s environments, and the statistical effects of environments depend on people’s genes. Only meaning (d) refers to a statistical interaction in the standard multiple regression or analysis of variance sense.

Two points are worth noting here. First, psychologists routinely confuse meanings (a) and (d). For example, when researchers write that “All reasonable scholars today agree that genes and environment interact to determine complex cognitive outcomes” (Bates et al., 1998, p. 195), some readers may assume that they are referring to the standard statistical meaning of the term “interaction,” (McClelland and Judd, 1993), i.e., a multiplicative rather than additive relation between variables, such as that between genetic and environmental influences. Instead, in this case the authors appear to be saying only that both genes and environment play a role in cognitive outcomes, a scenario that does not require a multiplicative relation between genes and environment. Second, meanings (c) and (d) are logically incompatible, because if the effects of genes and environment are not separable, then clearly they cannot be distinguished in statistical designs. The bottom line: when authors use the term “interaction,” they should be explicit about which of the four meanings they intend.

**(37) Medical model**. Although many authors who invoke the term “medical model” presume that it refers to a single conceptualization (e.g., Mann and Himelein, 2008), it does not. Some authors insist that the term is so vague and unhelpful that we are better off without it (Meehl, 1995). Among other things, it has been wielded by various authors to mean (a) the assumption of a categorical rather than dimensional model of psychopathology; (b) an emphasis on underlying “disease” processes rather than on presenting signs and symptoms; (c) an emphasis on the biological etiology of psychopathology; (d) an emphasis on pathology rather than on health; (e) the assumption that mental disorders are better treated by medications and other somatic therapies than by psychotherapy; (f) the assumption that mental disorders are better treated by physicians than by psychologists; or (g) the belief that mentally ill individuals who engage in irresponsible behavior are not fully responsible for such behavior (see Blaney, 1975, 2015, for discussions). Similar semantic and conceptual ambiguities bedevil the term “disease model” when applied to addictions and most other psychological conditions (e.g., Graham, 2013).

**(38) Reductionism**. There may be no greater insult in psychological circles than to brand a colleague a “reductionist.” Indeed, merely accusing a fellow faculty member of “being reductionistic” is often an effective conversation-stopper at cocktail parties. The negative connotation attached to this term neglects the point, overlooked by many authors (e.g., Harris, 2015), that “reductionism” is not one approach. Robinson (1995) delineated multiple forms of reductionism, including (a) nominalistic reduction, i.e., reduction at the level of names (“A brain structure called the amygdala plays a key role in fear processing”); (b) nomological reduction, i.e., reduction at the level of scientific explanation (“The perception of edges is mediated in part by feature detection cells in the visual cortex”); and (c) ontological reduction, i.e., reduction by eliminating immaterial entities (“Neuroscientific data strongly suggest that there is no immaterial soul”).

More broadly, we can differentiate between two quite different brands of reductionism: constitutive and eliminative, the latter termed “greedy reductionism” by Dennett (1995). The constitutive reductionist believes merely that everything that is “mental” is ultimately material at some level, and that the “mind” is what the brain and rest of the central nervous system do. Constitutive reductionists (like nomological reductionists; Robinson, 1995), who appear to comprise an overwhelming majority of psychologists and neuroscientists, reject mind-body dualism, the claim that the mind is entirely separable from the brain. In contrast, eliminative reductionists go a large step further (Lilienfeld, 2007). They contend that the “mind” will eventually be *explained away* entirely by lower-level concepts derived from neuroscience, and that mentalist concepts, such as thoughts, motives, and emotions, will ultimately be rendered superfluous by neuroscientific explanations. For eliminative reductionists, the field of psychology will eventually be “gobbled up” by neuroscience. Although we do not attempt to adjudicate the dispute between constitutive and eliminative reductionists here, suffice it to say that “reductionism” does not carry a single meaning in psychology. As a result, psychologists who use “reductionist” as a handy term of opprobrium against their colleagues must be explicit about which form of reductionism they are invoking.

## Oxymorons

**(39) Hierarchical stepwise regression**. Hierarchical and stepwise multiple regression are entirely separate – and incompatible - procedures. Still, they are readily confused, because in hierarchical regression, variables are entered in sequential steps. Specifically, in hierarchical multiple regression the investigator specifies an *a priori* order of entry of the variables, ideally on theoretical grounds. In contrast, in stepwise multiple regression, the investigators allows the computer to select the order of entry of the variables (and the final variables in the equation) on empirical grounds, namely, by choosing each successive predictor based on the highest incremental contribution to variability in the outcome variable (Wampold and Freund, 1987; Petrocelli, 2003). Many authors have wisely warned against the routine use of stepwise regression procedures on the grounds that they typically capitalize heavily on chance fluctuations in datasets and rarely yield replicable results (Thompson, 1989).

**(40) Mind-body therapies**. The term “mind-body therapy” (e.g., Naliboff et al., 2008) refers to a panoply of treatments, such as relaxation, meditation, Reiki, yoga, and biofeedback, that purportedly harness mental functioning to enhance physical health (Wolsko et al., 2004). This term implies erroneously that the “mind” is materially separate from the “body” and thereby endorses a simplistic version of mind-body dualism. Rather than conceptualizing such interventions as making use of the mind to influence the body, we should conceptualize them as making use of one part of the body to influence another.

**(41) Observable symptom**. This term, which appears in nearly 700 manuscripts according to *Google Scholar*, conflates signs with symptoms. Signs are observable features of a disorder; symptoms are unobservable features of a disorder that can only be reported by patients (Lilienfeld et al., 2013; Kraft and Keeley, 2015). Symptoms are by definition unobservable.

**(42) Personality type**. Although typologies have a lengthy history in personality psychology harkening back to the writings of the Roman physician Galen and later, Swiss psychiatrist Carl Jung, the assertion that personality traits fall into distinct categories (e.g., introvert vs. extravert) has received minimal scientific support. Taxometric studies consistently suggest that normal-range personality traits, such as extraversion and impulsivity, are underpinned by dimensions rather than taxa, that is, categories in nature (Haslam et al., 2012). With the possible exception of schizotypal personality disorder (but see Ahmed et al., 2013), the same conclusion holds for personality disorders (Haslam et al., 2012). Hence, if authors elect to use the phrase “personality type,” they should qualify it by noting that the evidence for a genuine typology (i.e., a qualitative difference from normality) is in almost all cases negligible within the personality domain.

**(43) Prevalence of trait X**. Authors in the psychological and psychiatric literatures frequently refer to “the prevalence” or “base rate” of attributes that are dimensionally distributed in the population, such as personality traits and intelligence. For example, one author team referred to the “greater prevalence of extraversion in American students” (p. 1153) compared with Korean students (Song and Kwon, 2012). Nevertheless, such terms as “prevalence,” “incidence,” “base rate,” “false positive,” and “false negative” are premised on a taxonic model: they presume that the phenomena in question are inherently categorical, that is, either present or absent in nature. For psychological features that are continuously distributed, such terms should be avoided. In the aforementioned phrase, referring to “higher levels of extraversion in American students” would have been more accurate.

**(44) Principal components factor analysis**. According to *Google Scholar*, this phrase appears in thousands of articles, including one co-authored by the first author of this manuscript (Reynolds et al., 1988). Nevertheless, this phrase is incoherent, because principal components analysis (which is commonly misspelled as “principle components analysis”) and factor analysis are incompatible approaches to data analysis. Principal components analysis is a data reduction technique that relies on the total variance of the variables in a dataset; its principal goal is to create a smaller set of weighted variables (variates) that approximate the variance of the original variables (Weiss, 1970). In contrast, factor analysis relies only on the shared variance of the variables in a dataset, and it is designed to identify underlying *dimensions* that best explain the covariation among these variables (Bryant and Yarnold, 1995). In contrast to principal components analysis, whose primary aim is to simplify a dataset by yielding fewer observed variables, the primary aim of exploratory factor analysis is to identify dimensions that ostensibly account for the covariation among the observed variables.

**(45) Scientific proof**. The concepts of “proof” and “confirmation” are incompatible with science, which by its very nature is provisional and self-correcting (McComas, 1996). Hence, it is understandable why Popper (1959) preferred the term “corroboration” to “confirmation,” as all theories can in principle be overturned by new evidence. Nor is the evidence for scientific theories dichotomous; theories virtually always vary in their degree of corroboration. As a consequence, no theory in science, including psychological science, should be regarded as strictly proven. Proofs should be confined to the pages of mathematics textbooks and journals (Kanazawa, 2008).

## Pleonasms

**(46) Biological and environmental influences**. This phrase implies that biological influences are necessarily genetic, and cannot be environmental. Nevertheless, “environmental influences” encompass everything external to the organism that affects its behavior following its fertilization as a zygote. As a consequence, the environment comprises not only psychosocial influences, but also non-genetic biological influences, such as nutrition, viruses, and exposure to lead and other toxins (e.g., Nisbett et al., 2012). The phrase “biological and environmental influences” is therefore a partial pleonasm.

**(47) Empirical data**. “Empirical” means based on observation or experience. As a consequence, with the possible exception of information derived from archival sources, all psychological data are empirical (what would “non-empirical” psychological data look like?). Some of the confusion probably stems from the erroneous equation of “empirical” with “experimental” or “quantitative.” Data derived from informal observations, such as non-quantified impressions collected during a psychotherapy session, are also empirical. If writers wish to distinguish numerical data from other sources of data, they should simply call them “quantified data.”

**(48) Latent construct**. A “construct” in psychology is a hypothesized attribute of individuals that cannot be directly observed, such as general intelligence, extraversion, or schizophrenia (Cronbach and Meehl, 1955; Messick, 1987). Therefore, all constructs are latent. The same terminological consideration applies to the phrase “hypothetical construct.” Authors would be better advised to instead use “construct” or “latent variable.”

**(49) Mental telepathy**. Telepathy, one of the three ostensible types of extrasensory perception (along with clairvoyance and precognition), is the purported ability to read other’s minds by means of psychic powers (Hyman, 1995). Hence, all telepathy is necessarily mental. The term “mental telepathy,” which appears to be in common currency in the academic literature (e.g., Lüthi, 2013; Sagi-Schwartz et al., 2014), implies erroneously that there are “non-mental” forms of telepathy.

**(50) Neurocognition**. Many authors have invoked the term “neurocognition” to refer to cognition, especially when conceptualized within a biological framework (e.g., Mesholam-Gately et al., 2009). Nevertheless, because all cognition is necessarily neural at some level of analysis, the simpler term “cognition” will do. In fairness, “neurocognition” is merely one among dozens of terms preceded by the prefix “neuro” that have recently become popular, including neuroeducation, neuroaesthetics, neuropolitics, neuropsychoanalysis, and neurosexology (Satel and Lilienfeld, 2013). In the words of one psychologist, “Unable to persuade others about your viewpoint? Take a Neuro-Prefix – influence grows or your money back” (Laws, 2012).

## Concluding Thoughts

We modestly hope that our admittedly selective list of 50 terms to avoid will become recommended, if not required, reading for students, instructors, and researchers in psychology, psychiatry, and similar disciplines. Although jargon has a crucial place in these fields, it must be used with care, as the imprecise use of terminology can engender conceptual confusion. At the very least, we hope that our article encourages further discussion regarding the vital importance of clear writing and clear thinking in science, and underscores the point that clarity in writing and thinking are intimately linked. Clear writing fosters clear thinking, and confused writing fosters confused thinking. In the words of author McCullough (2002), “Writing is thinking. To write well is to think clearly. That’s why it’s so hard.”

## Conflict of Interest Statement

The authors declare that the research was conducted in the absence of any commercial or financial relationships that could be construed as a potential conflict of interest.
